# Collagen-Based Scaffolds for Chronic Skin Wound Treatment

**DOI:** 10.3390/gels10020137

**Published:** 2024-02-08

**Authors:** Francesco La Monica, Simona Campora, Giulio Ghersi

**Affiliations:** Department of Biological, Chemical and Pharmaceutical Sciences and Technologies (STEBICEF), University of Palermo, Viale delle Scienze, Ed. 16, 90128 Palermo, Italy; francesco.lamonica03@unipa.it (F.L.M.); simona.campora@unipa.it (S.C.)

**Keywords:** wound healing, acute wound, chronic wound, scaffold, collagen, tissue engineering, regenerative medicine

## Abstract

Chronic wounds, commonly known as ulcers, represent a significant challenge to public health, impacting millions of individuals every year and imposing a significant financial burden on the global health system. Chronic wounds result from the interruption of the natural wound-healing process due to internal and/or external factors, resulting in slow or nonexistent recovery. Conventional medical approaches are often inadequate to deal with chronic wounds, necessitating the exploration of new methods to facilitate rapid and effective healing. In recent years, regenerative medicine and tissue engineering have emerged as promising avenues to encourage tissue regeneration. These approaches aim to achieve anatomical and functional restoration of the affected area through polymeric components, such as scaffolds or hydrogels. This review explores collagen-based biomaterials as potential therapeutic interventions for skin chronic wounds, specifically focusing on infective and diabetic ulcers. Hence, the different approaches described are classified on an action-mechanism basis. Understanding the issues preventing chronic wound healing and identifying effective therapeutic alternatives could indicate the best way to optimize therapeutic units and to promote more direct and efficient healing.

## 1. Introduction

Chronic skin wounds, known as ulcers, are a significant global health challenge, impacting millions of patients annually and imposing a considerable burden on their lives. Ulcers exhibit an extremely slow or absent healing rate, attributed to a complex interplay of internal and external factors [1]. The current therapeutic strategies employed by hospitals struggle to effectively regulate these factors, leading to prolonged hospital stays and huge costs. The gravity of this situation is exacerbated by the continual rise in risk factors associated with chronic wound development. Therefore, it is necessary to investigate therapeutic solutions able to restart the healing process by focusing on the specific mechanisms involved in wound healing. In recent decades, tissue engineering has gained prominence as a promising avenue, focusing on the development of functionalized skin substitutes capable of promoting wound healing, and reducing healing time [2]. Discovering an efficient therapeutic approach could be crucial in expediting the healing rate of chronic wounds, enhancing the quality of life for patients, and concurrently reducing healthcare costs. 

### Acute and Chronic Wound Healing

The largest human organ is the skin, which is the interface between the organism and the environment. Acting as a barrier for internal organs, the skin protects the organism against external agents and exploits its pivotal role in maintaining physiological homeostasis [3].

**Figure 1:**
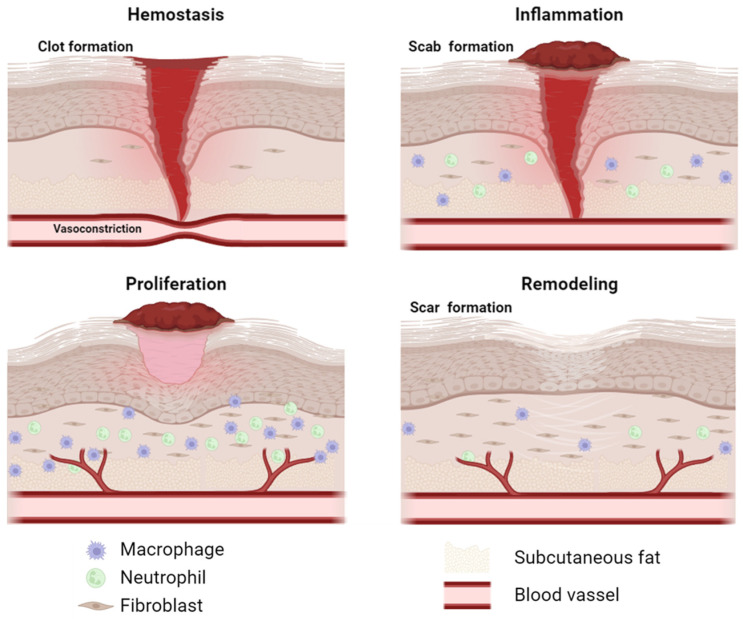
The wound-healing process. Created with BioRender.com.

**Figure 2:**
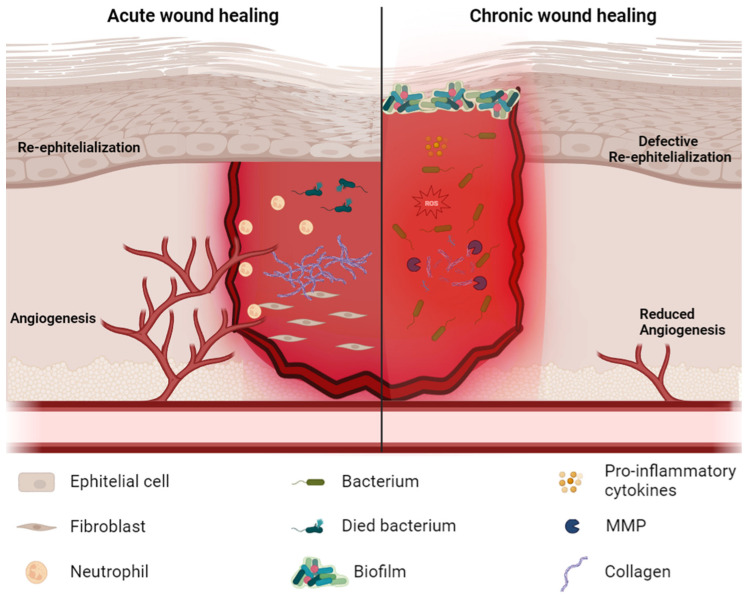
Acute and chronic wound healing. Created with BioRender.com.

However, the skin itself is subject to internal and external factors, that can physiologically interrupt its anatomical integrity and functionality, resulting in a wound [4,5]. Two categories of skin wounds can be distinguished, acute and chronic, which differ in the healing time [6,7]. Acute skin wounds form as a result of traumas that violate the integrity of the skin (although they can also affect the lower layers), due to both physical (e.g., sharp bodies) and environmental (e.g., chemicals, high temperatures, radiation, excessive pressure) agents [6,8]. Acute wounds heal independently through a physiological wound-healing process, restoring the affected region [7]. The healing rate of acute wounds depends both on factors specific to the wound, like size and depth, and the patient’s condition, such as age or pre-existing disease. Generally, actively healing wounds are free of bacterial infection and show cells in active fibroblast proliferation and secretion of extracellular matrix and molecules characteristic of inflammatory events such as proteases, metalloproteases, and pro-inflammatory cytokines [9] (Figure 1 and Figure 2). Wound healing is a complex, highly regulated process carried out by the resident fibroblasts, keratinocytes, and endothelial cells of the affected area, and immune cells to restore the anatomical and functional physiology of the wounded tissue [10,11,12]. 

Although it is a continuous process, wound healing is commonly divided into four overlapping phases: hemostasis, inflammation, proliferation, and remodeling (Figure 1). During the initial phase, blood flow is halted through both vasoconstriction and clot formation. Neutrophils are recruited to the wound first, followed by macrophages, to carry out the inflammatory phase aimed at eradicating bacteria entering the wound. In this phase, macrophages are classically activated (M1), secreting pro-inflammatory cytokines and stimulating inflammation. After a few days, bacteria and damaged tissue should have been removed, and the macrophage phenotype switches to the alternatively activated (M2), which secretes anti-inflammatory cytokines and growth factors to stimulate tissue regeneration. In three days, granulation tissue formation occurs, giving the wound bed a granular appearance due to the proliferation and migration of fibroblasts and the development of new blood vessels (angiogenesis). In this phase, fibroblasts begin to secrete collagen and other matrix molecules, facilitating wound filling and re-epithelialization. Finally, during the remodeling phase, the extracellular matrix undergoes modification by matrix metalloproteinases (MMPs), restructuring the tissue and forming the scar [7,13,14] (Figure 1). In healthy people, the wound-healing process takes place properly, without any interruption, to restore the original tissue structure and function. Each of these steps is essential for wound closure; however, certain pre-existing conditions and/or pathologies may lead to the interruption of one or more of these steps, slowing or interrupting the healing process and thus leading to chronic wound formation [10,15].

Wounds that do not heal spontaneously within a month are commonly called chronic wounds, also known as ulcers, characterized by a persistent inflammatory state [16,17]. Chronic wounds are a severe health problem that affects many people; it is estimated that 1–2% of the world population will be affected by ulcers at least once in their lifetime [18]. Moreover, ulcers determine a poor quality of life, causing health, social, and economic problems for patients and their families [18,19]. The increase in risk factors further worsens these statistics, with a higher number of patients affected by this problem each year. Age is an important determinant of chronic wounds; as individuals grow older, the risk of developing ulcers naturally escalates. Therefore, many studies have shown that the average age of people affected by ulcers is generally between 70 and 80 and probably these statistics will worsen due to the global increase in the elderly population [16,20,21]. One of the major risk factors for ulcers is comorbidity, a condition in which the patient affected by a wound already possesses one or more chronic underlying diseases [16,22]. Based on pre-existing chronic diseases, different types of ulcers can be distinguished. Conditions such as chronic venous insufficiency increase the risk of developing venous ulcers (VU), mainly affecting the extremities. Moreover, when a pathology forces the patient to stay in bed for a prolonged time, pressure ulcers (PU), also called decubitus wounds, are formed. A wound can become chronic if infected, due to the cells of the immune system being unable to kill bacteria. However, the most serious cases occur when a chronic wound coexists in a patient suffering from diabetes and/or obesity; diabetic ulcers almost always arise on the feet, hence the name diabetic foot ulcer (DFU) [16,17,23]. For this reason, most of the literature focused on finding appropriate care for diabetic and infected ulcers.

DFUs have a great impact both socially, as complications can worsen to the point of amputation, and economically [24]. The diabetic foot consists of deep tissue lesions associated with neurological disorders and peripheral vascular disease of the lower limbs [25]. The annual risk of contracting DFU for a diabetic patient is estimated to be 2.5% [26], while the overall prevalence of diabetic foot ulceration is 6.3%, higher in males (4.5%) than in females (3.5%), and higher in type 2 diabetic patients (6.4%) than in type 1 diabetics (5.5%) [24]. Moreover, the skin is our body’s first barrier against pathogens, and at the same time, it is rich soil for the growth of commensal bacteria that form microbiota [27]. Thus, when a wound forms, it is colonized by the bacteria present on the skin: it begins the inflammatory phase, where the body’s immune system responds immediately by eradicating bacterial contamination and preparing the wound for the next steps by recruiting signal cells and molecules that will allow tissue regeneration [28,29]. However, especially in patients with a compromised immune system, bacterial colonization may become critical to the point of overcoming the host’s immune system; there is a transition from colonization to infection, and the wound enters a continuous inflammatory phase, leading to wound chronicity. Among the bacteria most frequently found in ulcers, *Staphylococcus aureus* (93.5%), and *Pseudomonas aeruginosa* (52.2%) are particularly relevant, as they can form biofilms [28,29]. A biofilm is a bacterial community encapsulated within an extracellular polymeric substance (EPS) that nourishes and protects the bacterial community from antibiotics and antiseptics, rendering canonical treatments ineffective [30].

In chronic wounds, the environment is highly altered, with residing low-mitogenic cells that downregulate grow factor (GF) receptors and show an increasing level of reactive oxygen species (ROS) and metalloproteases (MMPs) that cause both GF decrease and extracellular matrix (ECM) degradation (Figure 2). These conditions do not allow ECM deposition and granulation tissue formation, originating a nonhealing wound [23]. Particularly, the inflammatory phase, the second stage of the wound-healing process, persists longer than usual in chronic wounds, due to factors such as hypoxia, prolonged trauma, the presence of bacteria, and compromised host responses [31]. Regardless of the etiological agent, a prolonged inflammation establishes an unfavorable environment for tissue regeneration. It attracts an increased influx of immune cells to the wound site, determining oxidative stress through the release of ROS and suppressing the release of antioxidants such as nitric oxide (NO). Oxidative stress reinforces the inflammatory response in a cyclic positive feedback loop by stimulating the release of MMPs (especially MMP-2 and MMP-9) and pro-inflammatory cytokines, leading to inflammation improvement, ECM degradation, and reduced GF levels. This context not only stops the healing process but also worsens the wound, increasing its susceptibility to infections and potentially culminating in necrosis and ulceration [31]. 

The management and treatment of chronic wounds represent a heavy burden on global health, accounting for about 3% of total expenditure. Overall, expenditure is estimated at USD 28.1 per year (in 2014), which increased to a maximum of 96.8 billion, considering wounds as secondary diagnosis [32,33]. The largest costs are incurred for diabetic wounds: for instance, it is estimated that in Europe the cost amount for DFU care is between EUR 4 and 6 billion per year and that the overall annual cost of a single DFU averages USD 8659 [24]. Costs increase considering the worldwide prevalence of diabetes mellitus and the longer life expectancy of diabetic patients [33]. 

The hospital management of chronic wounds is often a protracted and challenging process. A considerable aspect of treating chronic wounds is debridement, a procedure involving the removal of necrotic tissue present on the wound bed. This necrotic tissue serves as a fertile ground for bacterial proliferation, and its removal exposes well-perfused tissue with mitotically active cells, creating conditions conducive to wound closure [12,34]. Topical treatment employing antibacterial agents and antibiofilm substances is commonly employed to address infected wounds [35]. Additionally, the application of growth factors, which are often deficient in ulcerous conditions, is a widely used approach. Hyperbaric chambers, designed to counteract the hypoxia typical of ulcers, and negative pressure therapy are also utilized to promote the healing of ulcers [12,34]. While these methods facilitate the healing process, they are not absolute and may involve extended healing times. Wound dressings play a crucial role in wound care, primarily serving to shield wounds from the external environment, thereby preventing infections and maintaining optimal moisture levels. Moreover, dressings can be functionalized with additional substances such as antibiotics, antibiofilm agents, or growth factors to provide secondary therapeutic benefits. Depending on the material composition of the dressing, different effects can be achieved; for instance, hydrocolloids and hydrogels help maintain optimal moisture levels, thereby promoting autolytic debridement [36]. However, with the increasing prevalence of antibiotic resistance and the limited half-life of growth factors, the nonspecificity of these healing approaches, and, in general, the prolongation of healing times, require finding new approaches to treat ulcers, making the healing process faster and safer, reducing healthcare costs and improving patients’ quality of life.

In recent years, scientific research has shifted towards tissue engineering to overcome the limits of current chronic wound treatments. In this context, the use of biopolymeric functionalized scaffolds for regenerative medicine aims to stimulate and promote the healing process. The scaffold design and functionalization play a key role in developing the best medical strategy, depending on the nature and features of the wound. Specifically, this review focuses on therapeutic approaches analysis in using collagen-based scaffolds in combination with other biopolymers, for ulcer treatment. Notably, the scaffold project has to consider different aspects including inflammatory response, oxidative stress, hypoxia, cell proliferation, angiogenesis, and bacterial infections. Therefore, the review provides a comprehensive overview, highlighting the diverse strategies in the project and functionalization of collagen-based scaffolds for tissue engineering and regenerative medicine applications in ulcer treatment, focusing on the diabetic one and showcasing their potential across a broad spectrum.

## 2. Chronic Wounds Treatment

### Tissue Engineering for Wound Healing

One of the most important approaches in wound-healing treatment is tissue engineering, which aims to develop biological substitutes by applying engineering principles to restore wounded tissues [37]. The basis of tissue engineering is the creation of three-dimensional scaffolds engineering to anchor the cellular compounds and to release drugs in a stimuli-controlled way for the development of a newly forming tissue [2]. Regenerative medicine (RM) is strictly connected to tissue engineering as it employs tissue substitutes to “regenerate” damaged tissues, reinstating a physiological state both anatomically and functionally [38]. In clinical practice, regenerative medicine is currently integrated with standard care to attempt to reverse organ failure, thereby mitigating the potential need for transplantation. The advantage of regenerative medicine lies in its ability to tailor treatments for specific pathologies by introducing cells from the affected tissue and molecules to enhance targeted processes [39]. Scaffolds in regenerative medicine have multiple functions being biocompatible, degrading safely, and having pores for nutrient transport. Natural and synthetic polymers are commonly used as scaffolding materials because they are flexible, biocompatible, and biodegradable. Biomaterials are crucial for tissue repair as they provide a framework for cells and their attachment and can influence cellular activities like proliferation, differentiation, and tissue formation [2]. One of the most used biomaterials in tissue engineering and regenerative medicine is collagen.

Collagen, the main constituent of the ECM of connective tissues, represents approximately 30% of total mammalian proteins. Collagen plays a fundamental role in regulating the structural properties of bones, tendons, cartilage, and skin. There are more than 20 genetically distinct isoforms of collagen, of which collagens type-I and type-III are particularly present in dermal ECM, where they promote the penetration and proliferation of fibroblasts. These properties make collagen an excellent material, biocompatible and biodegradable, for tissue engineering and regenerative medicine applications. Furthermore, collagen, when polymerized, shows good mechanical properties and structural integrity, especially if copolymerized with other biopolymers [40,41,42]. Collagen scaffolds have been shown to allow exchanges of both gases and nutrients with the external environment, making them appropriate niches for cell growth [43]. In the context of wound healing, collagen scaffolds also play a fundamental role in regulating moisture levels and the activity of metalloproteases [44], becoming their substrate to prevent them from affecting the pre-existing on-growing matrix and providing collagen fibrils to reconstitute ECM.

Even if collagen as a biomaterial for scaffold design presents many advantages, it has some limitations. When polymerized in 3D structures, collagen is not structurally resistant and therefore it has a fast degradation rate, limiting its stability over time. The use of crosslinkers and copolymerization with other biomaterials can enhance structural properties and lifetime. Another limitation stems from the animal origin of collagen, frequently sourced from porcine or bovine, raising concerns about allergies, disease transmission (e.g., bovine spongiform encephalopathy), and bacterial contamination [45] Additionally, social and religious factors may hinder the acceptance of animal-derived collagen skin substitutes by some patients. An alternative option is marine-origin collagen, derived from sources like jellyfish or sponges [46]. Nevertheless, this alternative presents some negative features, including low biodegradation, amino acid content, and mechanical strength. Furthermore, synthetic collagen is challenging to produce due to its intricate structure [47,48,49]. In any case, in most cases, collagen remains the most used biomaterial in regenerative medicine.

In the last decades, collagen-based biomaterials in different forms have been widely used in regenerative medicine as a copolymer to create scaffolds and hydrogels in different therapeutic combinations for the treatment of wounds, including or no other factors, such as cellular components or bioactive molecules, to promote, improve and accelerate the healing process. One of the best-known cases is Integra^®^ Dermal Regeneration Template (IDRT), a membrane consisting of a layer of collagen type-I crosslinked to glycosaminoglycans (chondroitin-6-sulfate) covered with a semipermeable silicone sheet [50]. Integra today is used for the treatment of surgical wounds in the clinic, as it is approved for the treatment of venous ulcers and combat-related wounds. In general, it promotes cell migration, and angiogenesis, and allows for granulation tissue formation, but is susceptible to infection and graft loss [50,51]. In a further study, researchers have tried to enhance Integra’s healing power by adding microvascular fragments extracted from adipose tissue (ad-MVFs) [52]. The results showed rapid blood perfusion within 3–6 days after treatment, due to the enhanced angiogenic germinative activity of the ad-MVFs which, stimulated to release pro-angiogenic factors, were able to both perfuse the newly formed tissue and reconnect to the host vessels. The ability of collagen to enhance microvessel sprouting and anastomosis with the host circulatory system was further confirmed by a study in which ad-MVFs were seeded onto 3D collagen hydrogels, which were then used for transplantation of islets of Langerhans. Microvessels showed high expression of factors such as VEGF and PDGF, suggesting the activation of an angiogenic pathway that led to the proper vascularisation of the islets [53]. Over the years, many skin substitutes that exploit, like Integra, the beneficial potential of collagen to promote tissue regeneration, facilitating the healing of chronic wounds of different natures, have been engineered (Table 1) [54,55,56].

On these bases, this review aims to provide a complete vision of the main approaches in which collagen-based biomaterials have been used to solve the problem of chronic wounds. Particularly, studies are classified into different groups depending on the process they target to promote wound healing, as shown in Table 2.

## 3. Scaffold Properties for Wound-Healing Treatment

**Figure 3:**
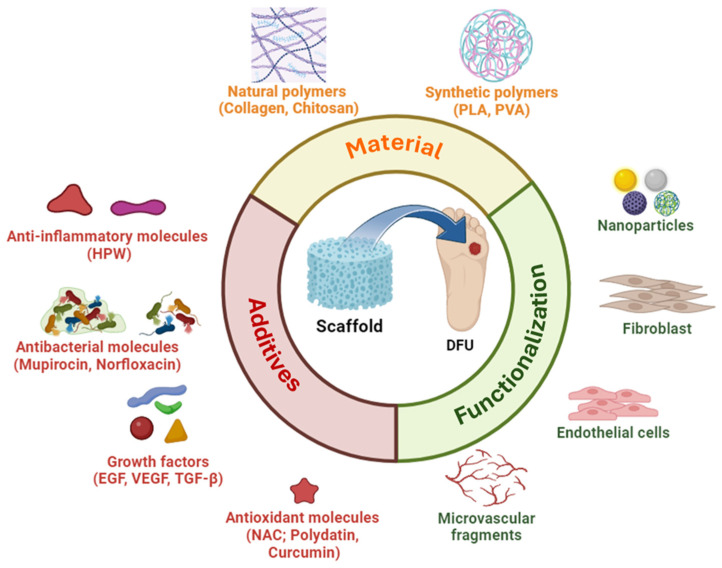
Scaffold design: material, functionalization, and additives. Created with BioRender.com.

Scaffold design plays a pivotal role in wound-healing treatment. Generally, they can be made of synthetic polymers combined with components of extracellular matrix, principally collagen (Figure 3). Depending on the functionalization (with cells, microvascular fragments, or nanoparticles) and the conjugation with additives like growth factors, antimicrobial, anti-inflammatory or antioxidant molecules, they can be involved in different applications concerning angiogenesis and revascularization, inflammation, oxidative stress, microbial infections antibacterial, antioxidant, and anti-inflammatory properties (Figure 3).

### 3.1. Promoting Angiogenesis and Revascularization

One of the key aspects of the wound-healing process is the stimulation of angiogenesis, especially in the case of diabetic foot ulcers (DFUs), characterized by hypoxia. The formation of a new capillary network at the wound site would ensure a constant supply of oxygen and nutrients, remove metabolic waste, rebalance pH, reduce levels of ROS, and, in general, promote healing. For this reason, many studies focused on creating wound dressings that could enhance wound healing by particularly boosting the angiogenic process. For instance, Tan and colleagues proposed a new composite biomaterial as a drug delivery system for the treatment of diabetic wounds induced in a rat model. They developed a collagen scaffold with CBD-VEGF, a fusion protein created by combining the vascular endothelial growth factor (VEGF) with a collagen-binding domain (CBD) to permit a gradual and sustained release of VEGF, one of the key pro-angiogenic factors crucial for wound healing (Figure 3). Compared to control groups, rats treated with this kind of scaffold showed an enhancement of the formation of new blood vessels, promoting cell migration and wound closure [57]. In the following study, pro-angiogenic properties were further enhanced by coupling another fusion protein to the CBD-VEGF fusion protein, CBD-SDF-1α (cell-derived factor 1α, known for its role in sending homing signals to endothelial progenitor cells, EPCs) [58,95]. The two factors were attached to a collagen scaffold and were gradually released into the surrounding environment when used to treat diabetic rat wound models. In vivo tests showed that the scaffold promoted angiogenesis and vessel reparation, reduced inflammation, and overall accelerated the healing of the diabetic wound by improving cell proliferation, re-epithelialization, and extracellular matrix accumulation [58]. On the other hand, Ding et al. investigated the ability of the anti-apoptotic factor Bcl-2, to enhance the survival of adipose-derived stem cells (ADSCs) and increase their expression of VEGF to promote angiogenesis [59]. Specifically, collagen-based Bcl-2-modified ADSCs (Bcl-2-ADSCs) scaffolds were synthesized to treat induced diabetic wound models in mice. The collagen–Bcl-2-ADSCs scaffold demonstrated significant efficacy in promoting tissue revascularization and accelerated growth compared to all controls, including treatment with collagen scaffold-ADSCs without Bcl-2, substantiating its therapeutic potential [59]. The angiogenic process can also be enhanced by combining fish collagen and copper/cobalt ion-loaded bioactive glass to create a microfibrous mat for the treatment of diabetic wounds as proposed by Jana and colleagues [60]. Both copper and cobalt promote angiogenesis through physical interaction and by inducing the transcription of many pro-angiogenic genes via the stabilization of hypoxia-inducible factor-1 (HIF1). The scaffold thus formed not only possesses good mechanical and cytocompatibility capabilities but has also demonstrated the ability to enhance wound healing in diabetic conditions, by promoting angiogenesis, extracellular matrix formation, and wound closure in in vivo rabbit models.

Collectively, these studies focus on implementing or stabilizing factors involved in the angiogenic process, promoting both angiogenesis and vasculogenesis, ultimately increasing the number of vessels and improving nutrient transport.

### 3.2. Counteracting Inflammation

The persistence of a chronic inflammatory state represents one of the main features of chronic wounds. This condition creates an environment within the wound that counters the regenerative process, not only providing a favorable setting for bacterial proliferation but also potentially leading to necrosis. The inflammatory process can be modulated in various ways, such as through the use of nanoparticles (Figure 3) [96]. For example, gold nanoparticles (AuNPs) can be employed, serving not only to impart greater mechanical stability to collagen scaffolds (CSs) crosslinked with glutaraldehyde but also to possess anti-inflammatory activity. AuNPs have been shown to downregulate the pro-inflammatory factors TNFα and interleukin-6 (IL-6) thereby suppressing inflammation [97]. In a full-thickness skin wound model, they exhibited promising therapeutic effects, including a reduced inflammatory response, improved wound closure, and enhanced tissue revascularization [61]. In other cases, molecules with anti-inflammatory activity can be coupled to scaffolds to target the inflammatory phase during wound healing. Anti-inflammatory agents, like thymosin beta 4 (Tβ4), have been explored for their inhibitory effects again NF-κB and inflammatory cytokines such as tumor necrosis factor-α (TNF-α), interleukin-1β (IL-1β), and IL-10 [98]. In addition, the Tβ4 activity was investigated also for its pro-angiogenic properties. Specifically, the study employs a porous collagen/chitosan scaffold containing Tβ4 to assess its therapeutic potential in the healing of diabetic rats with hindlimb ischemia. The results showed that the gradual release of Tβ4 from the scaffold inhibited the inflammatory process in favor of angiogenesis, resulting in faster closure of the diabetic wound and tissue with improved dermal reorganization and increased vascularization [62]. Another strategy aims to reduce inflammation by modulating MMP-9 activity, which is the principal MMP responsible for chronic inflammation, along with MMP-2 [63]. Specifically, pioglitazone-loaded lipidic nanoparticles have been incorporated into collagen–chitosan scaffolds to promote diabetic wound healing by reducing inflammation, owing to pioglitazone’s ability to inhibit MMP-9. In in vivo diabetic rat models, the scaffolds downregulated MMP-9 levels, leading to a lower inflammatory response and faster wound closure compared to controls. On the other hand, the expression of genes involved in the inflammation process can be downregulated through noncoding RNA. Specifically, a MMP-9 small interfering RNA (siRNA) was conjugated to a collagen–glycosaminoglycan scaffold, to obtain a drug delivery system capable of reducing MMP-9 expression in both dermal fibroblasts and M1 macrophages suggesting a potential therapeutic effect in modulating inflammation for in vivo use [64]. Antisense strategy was adopted also in another study in which collagen scaffolds were coated with the antisense oligodeoxynucleotides against connexin 43 (Cx43asODN), highly expressed in the foreign body (such as a scaffold) inflammatory reactions. In vitro testes have demonstrated the capability of inhibiting cell migration from the wound edge; while in vivo wound-healing assays showed improved re-epithelialization and reduced inflammation intensity, resulting in a more efficient wound closure [65,99].

### 3.3. Counteracting Oxidative Stress

The increased inflammation in the wound bed causes a rise in ROS, leading to oxidative stress and exacerbating inflammation by degrading the matrix and elevating levels of pro-inflammatory cytokines in the local environment. Many studies have focused the oxidative stress, trying to reduce it, and promoting wound healing. One of the most utilized antioxidant molecules is N-acetylcysteine (NAC), crucial for reducing excessive ROS production during inflammation, thereby promoting wound healing. Particularly, NAC is involved in glutathione formation, the most important biological antioxidant due to its ability to act as a free radical’s scavenger [100]. For example, two different types of sandwich scaffolds were created as delivery systems for N-acetylcysteine (NAC). In both scaffolds, the limited mechanical properties of collagen were complemented by superior structural polymers, like polycaprolactone (PCL) [66] or polyamide [67], which enhanced the mechanical properties. In the first case, a PCL core was complemented by NAC-collagen on both sides, while in the second one, an inner electrospun polyamide layer was surrounded by collagen/NAC. When tested in in vivo rat models for their ability to promote wound healing, both scaffolds demonstrated to increment wound healing compared to controls, improving collagen deposition, revascularization, and wound re-epithelialization. Moreover, NAC can be used as an antioxidant agent not only against reactive oxygen species but also against graphene oxide (GO). Indeed, a NAC-loaded graphene oxide–collagen hybrid membrane (N-Col-GO) for enhanced skin regeneration was synthesized [68]. The scaffold exhibited features such as water retention and biocompatibility. In mouse models of full-thickness skin wounds, the membrane promoted the reduction in ROS during the inflammatory phase, consequently facilitating healing by supporting various processes, including angiogenesis, re-epithelialization, and cell migration. Furthermore, the membrane showed anti-fibrotic effects, preventing the formation of hypertrophic scarring. Also, the polyphenol polydatin possesses antioxidant activity similar to NAC, by seizing free radicals, consequently reducing the amount of ROS [101]. A collagen scaffold conjugated with polydatin was employed to enhance wound healing in both diabetic and nondiabetic rat models [69]. The results for both types of ulcers were very encouraging, showing antioxidant ability higher 82% than untreated controls. Overall, the construct showed accelerated wound closure, and improved angiogenesis, epithelialization, and collagen deposition. Many molecules, like curcumin, possess both antioxidant and anti-inflammatory activities, mainly because these two processes are interconnected. Topical curcumin treatments in diabetic-rat-induced wounds manifested reduced inflammation by downregulation of pro-inflammatory factors such as TNF-α, IL-1β, and MMP-9, and upregulation of IL-10. Moreover, antioxidant activity was importantly increased by the overexpression of enzymes such as superoxide dismutase, catalase, and glutathione peroxidase [102]. On the other hand, the properties of collagen/chitosan scaffolds can be enhanced by adding curcumin-conjugated nanoparticles (CNs) to treat full-thickness wounds in Wistar rats, as proposed by Rezaii and colleagues [70]. Due to curcumin’s antioxidant and anti-inflammatory capabilities, along with its ability to increase the expression levels of the crucial factor in the wound-healing process TGF-β1 (transforming growth factor- β1), a significant acceleration in the healing rate was achieved in the treated rats compared to the controls. This was evidenced by a significant reduction in wound area, increased epidermal thickness, augmented density of granulation tissue, elevated number of new vessels, and a higher collagen content. In another study, copolymeric alginate/collagen scaffolds coupled to curcumin-conjugated chitosan nanoparticles (CUR-CSNPs) were used to treat diabetic wounds [71]. The nanohybrid scaffolds showed optimal physical qualities, corrosion rate, and biodegradation and ensured a sustained release of curcumin. Wounds induced in diabetic mouse models and treated with the scaffolds allowed significantly faster wound contraction than in the control groups. In addition, histopathological analysis revealed increased fibroblast proliferation, collagen synthesis, and orderly dermal formation, while reducing chronic inflammation.

### 3.4. Counteracting Microbial Infections

Bacterial contamination, as previously discussed, represents one of the main etiological agents in chronic wounds, particularly when biofilm formation occurs. Biofilms shield bacteria from both the host’s immune system and externally introduced molecules with antibacterial activity [103]. Additionally, the increasing antibiotic resistance of some bacterial strains makes it essential to identify new molecules potentially suitable for combating infections. Many studies combine the beneficial properties of scaffolds with the action of antibiotics, natural extracts, or nanoparticles with antibacterial activity [104]. For instance, bio-nano scaffolds were manufactured with a PVA (polyvinyl alcohol) core and an outer layer of collagen [72]. Both layers were incorporated with *Licorice* extracts, a traditional herb containing molecules with antibacterial and antiviral properties. In vivo studies involving full-thickness wounds induced in rabbits demonstrated tissue regeneration promotion within 10 days, enhancing wound healing. On this basis, the intrinsic antibacterial potential of silver in the form of silver sulfadiazine was conjugated with a nanofibrous copolymeric scaffold composed of collagen, ethyl cellulose (EC), and poly-lactic acid (PLA) [73]. In vitro studies suggested a good biocompatibility and a nontoxic effect. However, the rates of cell adhesion and proliferation, along with the antibacterial potential, were examined and demonstrated satisfactory results, making this scaffold a potential candidate for wound healing. On the other hand, the antibacterial power of mupirocin, coupled with silica microspheres, was investigated to enhance the healing properties of a collagen scaffold for chronic wounds [74]. In vivo studies highlighted a faster closure of wounds, attributed to the shortened duration of the inflammatory phase due to the presence of mupirocin, increased fibroblast proliferation, and subsequent enhanced collagen deposition. Moreover, Indrakumar et al., coupled collagen properties with those of molybdenum trioxide nanoparticles, known for their antibacterial and pro-angiogenic activities, to investigate their wound-healing potential [75]. Using in vivo wound models in Wistar rats, the composite scaffolds demonstrated a faster wound closure compared to the untreated controls. Additionally, the nanoparticles exhibited both antibacterial and pro-angiogenic activities, even at low concentrations. These findings were confirmed by histological sections, revealing improved revascularization, collagen deposition, and overall tissue reformation, including skin appendages [75].

Chitosan, one of the most commonly used copolymers with collagen, is often chosen for its ability to enhance the structural properties of collagen and for its antimicrobial and antioxidant properties [105]. Chitosan is a polysaccharide biomaterial derived from chitin, exhibiting antimicrobial properties against a broad spectrum of bacteria, both Gram-positive and Gram-negative [106]. On this basis, the antibacterial properties were particularly emphasized by combining the antibiotic activity of Norfloxacin with the intrinsic antibacterial properties of chitosan [76]. Particularly, chitosan was copolymerized with collagen to create an antibiotic-medicated collagen/chitosan sponge scaffold for skin tissue engineering. In vivo, tests on full-thickness wound models in rats demonstrated that both the norfloxacin-treated and the untreated scaffolds accelerated healing compared to the controls. However, the former exhibited superior properties with reduced inflammation and faster wound closure. Histological tests further confirmed the accelerated healing rate of the antibiotic-coupled scaffold, resulting in faster tissue regeneration, a normal intact epidermal layer, and decreased inflammatory cell infiltration.

### 3.5. Scaffoldings with Antibacterial, Antioxidant, and Anti-Inflammatory Properties

To improve the healing properties in chronic wounds treatment, scaffolds can be combined with natural extracts, a blend of bioactive molecules that collectively possess various abilities, including antioxidant, anti-inflammatory, and antibacterial properties. Often, underlying action mechanisms of the single molecules are not well known, and only the therapeutic power of the partial or total extract is studied. For instance, scaffolds made of a mix of collagen from bovine and marine sources were synthesized and functionalized alternately or in combination with two plant extracts: extracts from *Pistacia lentiscus* (with antioxidant and antibacterial properties) and from *Calendula officinalis* (known for its therapeutic potential against skin diseases) [77]. These scaffolds, when soaked with bioactive extracts, significantly enhanced wound closure in treated mice compared to conventional gauze, reducing the healing period of four days (from 13 to 9), especially those soaked with *Pistacia lentiscus* extracts, which showed a 50% wound closure at 3 days. Alternatively, honey-propolis wax (HPW), a resinous mixture produced by honeybees with antimicrobial, anti-inflammatory, and antioxidant properties, was used as a matrix to host collagen hydrolysates for wound treatment [78]. HPW increased re-epithelialization and wound closure speed in induced wounds in in vivo murine models. Specifically, the resin inhibited the infiltration of immune cells, reducing inflammation, as confirmed by the downregulation of pro-inflammatory markers such as IL-1β (interleukin-1β) and TNF-α (tumor necrosis factor- α). Conversely, the expression of genes involved in extracellular matrix formation, such as VEGF, EGF (epidermal growth factor), and TGF-β, have been upregulated, promoting the regenerative process. In a different approach, a collagen scaffold was fabricated, incorporating chitosan microspheres conjugated with mupirocin (M-CSM) on one side and Piper betle (PB) Linn leaf extracts on the other [79]. PB leaves are known for their anti-inflammatory, antioxidant, antidiabetic, and antimicrobial properties. The intrinsic antibacterial activities of chitosan, mupirocin, and PB extracts provide the construct with triple antibacterial action, in addition to other unique properties attributed to the molecules within PB extracts. In vivo tests conducted on a full-thickness wound model in rats demonstrated an accelerated regenerative process, characterized by increased collagen deposition, fibroblast proliferation, blood vessel formation, and reduced inflammation. In another study, wheatgrass, recognized for its bioactive components with anti-inflammatory, antibacterial, and antioxidant properties, has been used as a crosslinker for collagen, to augment the stability and therapeutic attributes of the resulting aerogel [80]. In in vitro tests, the aerogel supported cell proliferation and exhibited notable antibacterial efficacy, with a concentration-dependent antibacterial impact against Gram-positive bacteria. In a rat in vivo wound-healing model, the construct displayed enhanced proangiogenic activity, contributing to an accelerated wound-healing process. Also, *Acanthus ebracteatus vahl* extracts, known for their potent anti-inflammatory, antibacterial, and antioxidant properties, were administered topically daily, combined with a collagen scaffold in a mouse model of full-thickness wounds [81]. Results underline the superior effectiveness of the synergistic action between collagen and *Acanthus ebracteatus vahl* extract compared to either component alone in fostering wound closure. The complete treatment demonstrated a reduction in inflammation by limiting neutrophil infiltration, facilitated angiogenesis through elevated production of VEGF, and expedited re-epithelialization.

### 3.6. Promoting Cell Proliferation

Although antibacterial, antioxidant, and anti-inflammatory properties play a pivotal role in scaffold design for wound-healing treatment, another very important aspect focuses on improving cellular proliferation, specifically on fibroblasts (cells predominantly found in the dermis). Several approaches have been employed to achieve this objective. For example, the combination of plasma pretreatment and kefiran coating on PLA (poly-lactic acid) electrospun scaffolds can increase fibroblast cell attachment and proliferation, and collagen production [82]. On the other hand, another strategy harnesses the fibroblast-proliferating and apoptosis-inhibiting properties of soluble silkworm gland hydrolysate (SSGH), combining it with human collagen within a scaffold designed for treating full-thickness excisional wound models [83]. In vivo outcomes have demonstrated that SSGH/collagen scaffolds expedited wound healing compared to control groups, stimulating cells to generate essential growth factors, thereby amplifying capillary and fibroblast proliferation. In another study collagen/PLGA/chitosan nanofibers containing nanoparticles loaded with growth factors such as VEGF and basic fibroblast growth factor (bFGF) were electrospun [84]. Both growth factors assume a crucial role during wound healing, by stimulating cell proliferation and angiogenesis. In in vivo studies in diabetic wounded mice models, the scaffold showed improved wound closure, angiogenesis, fibroblast proliferation, collagen deposition, and re-epithelialization. In another investigation, the stimulation of cellular proliferation was achieved through the incorporation of autologous micrografts of full-thickness dermal tissue into a collagen–glycosaminoglycan scaffold [85]. As these micrografts originate from healthy tissue, they serve as nucleation centers for cellular proliferation, emitting signals (pro-angiogenic and growth factors, or ECM molecules) that stimulate cells in the wound bed to multiply. In murine dorsal wound models, the scaffolds expedited the healing process by fostering collagen deposition, cellular migration, and proliferation, while enhancing vascularization.

In some instances, the promotion of cell proliferation is complemented by antibacterial activity. In this investigation, *Aloe vera*, recognized for its capabilities in wound hydration and in stimulating fibroblast proliferation and collagen synthesis, was integrated into the matrix constituted of Zein/PCL (zein/poly-ε-caprolactone) and collagen and supplemented with ZnO nanoparticles (NPs) for antibacterial efficacy [86]. The nanofibrous mats demonstrated robust antibacterial characteristics and facilitated the proliferation, adhesion, and growth of cells. In another study, a bilayer scaffold was developed, consisting of mixtures of gelatin and collagen electrospinned onto a porous chitosan scaffold and containing *Lithosperm radix* (LR) extract, which has resistance to bacterial contamination and promotes fibroblast viability and proliferation. In vivo experiments on rats showed that the bilayer scaffold containing LR extract had the highest wound recovery rate compared to other dressings [87]. An improvement in the therapeutic effects of human umbilical cord mesenchymal stem cells (hUC-MSCs) in wound healing was obtained by utilizing an in situ injectable hydrogel composed of sodium alginate (SA) and collagen type-I (Col) [88]. SA/Col hydrogel loaded with hUC-MSCs significantly promotes wound closure by enhancing cell proliferation, re-epithelialization capability, collagen deposition, and tissue remodeling. Moreover, the hydrogel mitigates inflammation and promotes angiogenesis by increasing the secretion of proangiogenic factors such as VEGF and TGF-β1.

Therefore, cell proliferation has been promoted both directly providing growth factors and stimulating their production using natural extracts or health-tissue components. Overall, fibroblast proliferation is promoted by improving the wound-healing rate.

### 3.7. Stimulating ECM Regeneration

Different studies have focused on promoting the regeneration of the extracellular matrix, both using matrix molecules for scaffold creation and by designing architectural environments that mimic the ECM, providing a physiological, favorable environment for tissue regeneration. For instance, the inflammation and the ECM were modulated by incorporating doxycycline into a collagen/chitosan scaffold [89]. Doxycycline is a specific inhibitor for matrix metalloproteinase 9 (MMP-9) involved in the inflammatory phase for matrix remodeling during physiologic wound healing. However, in chronic contexts, such as diabetic ulcers, the excessive presence of MMPs, particularly MMP-9, can disrupt the ECM, further delaying the healing process. Diabetic wound models demonstrated faster and more complete healing compared to controls, with reduced levels of both inflammation and infection when treated with the doxycycline scaffold. To regulate the balance between extracellular matrix degradation and neoformation, Natarajan and Kiran proposed a system aimed at modulating enzymatic activity through juglone, a quinone-based nutraceutical that improves collagen scaffolds conferring anti-proteolytic and pro-angiogenic characteristics when combined with silver nanoparticles [90]. Specifically, a collagen scaffold was engineered as a drug delivery system for silver nanoparticles conjugated with juglone. In in vivo wound-healing experiments using Wistar rats, the system was found to promote the angiogenic process through the regulation of MMPs. The scaffold improved the wound-healing process, exhibiting a higher wound closure rate than controls, probably due to the increased juglone-induced angiogenesis [90]. In another approach, an engineered scaffold is proposed to emulate the dermal architecture using collagen type-I and tropoelastin as copolymers to promote wound healing [91]. The scaffold exhibited a functional stratum corneum, improved re-epithelialization, and the presence of elastin in the dermis, not typically present in wound healing. An improvement in mimicking the skin’s architecture was obtained by creating a bilayer scaffold consisting of a collagen/chitosan film to emulate the epidermis and a collagen–glycosaminoglycan scaffold to simulate the dermis features [92]. In vitro tests revealed that the epidermal layer exhibited not only antibacterial properties but also the ability to support keratinocyte proliferation for epidermal reformation. On the other hand, the dermal layer promoted endothelial cell proliferation, suggesting a role in enhancing revascularization. Furthermore, the organization of collagen type-I and type-III fibers have been modulated in a specific ratio to create a biomimetic scaffold with a crossed-fiber organization [93]. Compared to scaffolds featuring aligned and randomly arranged fibers, in in vitro tests the crossed collagen nanofibrous scaffolds induced a distinct response in fibroblasts concerning morphology, migration, and gene expression related to wound healing. Moreover, despite all three scaffold types enhanced the regenerative process, in vivo tests on diabetic rats showed that the crossed nanofibrous scaffolds yielded the best results; indeed, they reduced inflammation and promoted angiogenesis and cell migration.

### 3.8. Particular Case

In some cases, collagen structures can be functionalized to counteract the underlying disease that causes ulcer formation. For instance, in this scenario, Glucophage (metformin), an anti-diabetic agent, was used and linked to collagen/PLGA (poly-lactic-co-glycolic acid) membranes for treating diabetic ulcers [94]. Metformin was chosen for its ability to promote re-epithelialization and prevent collagen degradation. These composite membranes were tested in in vivo wound-healing assays in streptozotocin-induced diabetes rats. The results showed that the release of high concentrations of metformin ensured faster healing of diabetic wounds, with improvements both in collagen content and the re-epithelialization process compared to collagen/PLGA nanofibers alone and gauze dressings.

## 4. Conclusions

Chronic wounds or ulcers, which are wounds that do not naturally heal, pose a significant global health challenge due to their widespread occurrence, increased risk factors, and negative impact on lifestyle. The current treatments available are not universally effective and often fall short of ensuring complete healing. Additionally, even when successful, these treatments significantly contribute to global healthcare costs due to prolonged healing periods. In recent years, there has been a notable increase in studies focusing on the exploration of alternative, faster, and more effective healing methods utilizing tissue engineering and regenerative medicine. The primary objective is to develop biopolymeric three-dimensional structures, either combined or not with molecules possessing diverse properties, to expedite the wound-healing process. Notably, collagen has emerged as one of the most extensively utilized biopolymers, thanks to its exceptional biocompatibility, biodegradability, and inherent ability to promote wound healing. This review aims to systematically gather findings from regenerative medicine studies that, through comprehensive in vitro and in vivo testing, have demonstrated the practical applicability of collagen-based structures in the context of wound healing. The therapeutic approaches discussed are categorized based on the specific biological processes they target to facilitate and accelerate the healing of wounds. Consequently, these scaffold-based approaches can be tailored to exhibit specific therapeutic properties based on the biopolymers constituting them and on the strategic conjugation of various molecules, growth factors, or nanoparticles.

The review explores the therapeutic effectiveness of collagen as a biomaterial in tissue engineering and highlights critical processes that must be addressed to reactivate the wound-healing process in nonresponsive wounds. As discussed, chronic wounds are characterized by an excessive inflammatory response, oxidative stress, hypoxia, and susceptibility to bacterial infections, potentially leading to the formation of biofilms. Therefore, tissue engineering represents a promising strategy for ulcer treatment by using smart polymers carrying molecules, cellular components, nanoparticles, and other elements. The goal is to optimize the healing process by tailoring specific interventions for each type of ulcer. Numerous studies explore different biopolymers conjugated with both natural and synthetic molecules, making the identification of singular therapeutic solutions a complex task. Consequently, an in-depth analysis of therapeutic constructs presented in the literature becomes necessary to identify biomaterials, molecules, and other components with the most significant impact on wound healing to be strategically combined to create an ideal skin substitute that effectively counteracts the underlying processes associated with each category of chronic wounds.

Future studies should focus on combining different compounds in a single therapeutic unit to counter chronic wounds on multiple fronts. Anti-inflammatory and antioxidant activities should be coupled to convert the reducing and hypoxic chronic wound bed into a microenvironment that allows wound healing. At the same time, cell proliferation, angiogenesis, and ECM molecule accumulation must be stimulated to promote wound closure and scar formation. Incorporating antibacterial and anti-biofilm molecules into scaffolds allows safer wound healing by preventing bacterial contamination, consequently expediting the inflammation phase. A complete skin substitute carrying all these features would be a promising alternative in chronic wound treatments due to its ability to promote wound healing from different points of view.

## Figures and Tables

**Table 1:** Collagen-based FDA-approved skin substitutes.

Name	Composition	Use
OrCel^®^	Bilayered bovine collagen scaffold seeded with both keratinocytes and fibroblasts	Burns
Biobrane^®^	A porous nylon mesh with a silicone membrane including a porcine type I collagen dermal matrix	Chronic wounds, venous ulcers, partial thickness burns, dermabrasion
Apligraf^®^	Bilayered construct composed of an upper part with keratinocytes embedded on a bovine collagen type I gel and a lower part with fibroblasts included into a dermal matrix	Burns, and pressure, venous, and diabetic ulcers

**Table 2:** Application of different types of scaffolds for wound-healing treatment.

Application	Scaffold	Functionalization	Ref.
Promoting angiogenesis and revascularization	Collagen scaffold	CBD-VEGF fusion protein	[57]
	Collagen scaffold	CBD-VEGF and CBD-SDF-1α fusion proteins	[58]
	Collagen scaffold	Bcl-2-ADSCs	[59]
	Fish collagen and bioactive glass microfibrous mat	Copper/cobalt ion	[60]
Counteracting inflammation	Crosslinked collagen scaffolds	Gold nanoparticles	[61]
	Collagen/chitosan scaffold	Thymosin beta 4	[62]
	Collagen/chitosan scaffold	Pioglitazone	[63]
	Collagen/GAG scaffold	siMMP-9	[64]
	Collagen scaffold	Antisense oligonucleotides against Cx43	[65]
Counteracting oxidative stress	Collagen–polycaprolactone sandwich scaffold	N-acetylcysteine	[66]
	Collagen–polyamide sandwich scaffold	N-acetylcysteine	[67]
	Graphene oxide–collagen hybrid membrane	N-acetylcysteine	[68]
	Collagen scaffold	Polydatin	[69]
	Collagen/chitosan scaffolds	Curcumin-conjugated nanoparticles	[70]
	Alginate/collagen scaffolds	Curcumin-conjugated chitosan nanoparticles	[71]
Counteracting microbial infections	PVA (Polyvinyl alcohol) and collagen scaffold	*Licorice* extracts	[72]
	collagen, ethyl cellulose (EC), and poly-lactic acid (PLA) scaffold	Silver Sulfadiazine	[73]
	Collagen scaffold	Mupirocin-loaded silica microspheres	[74]
	Collagen scaffold	Molybdenum trioxide nanoparticles	[75]
	Collagen/chitosan scaffold	Norfloxacin	[76]
Combination of antibacterial, antioxidant, and anti-inflammatory properties	Scaffolds made of a mix of bovine and marine collagen	*Pistacia lentiscus* and/or *Calendula officinalis*	[77]
	Honey-propolis wax	Collagen hydrolysates	[78]
	Collagen Scaffold	Mupirocin-Loaded Chitosan Microspheres and Piper Betle Extract	[79]
	Collagen aerogel	Wheatgrass	[80]
	Collagen scaffold	*Acanthus ebracteatus vahl* extract	[81]
Promoting cell proliferation	PLA scaffold	Plasma pretreatment and kefiran coating	[82]
	Collagen scaffold	Silkworm gland hydrolysate (SSGH)	[83]
	Collagen/PLGA/chitosan scaffold	VEGF and bFGF	[84]
	Collagen–glycosaminoglycan scaffold	Autologous micrografts	[85]
	Zein/PCL (zein/poly-ε-caprolactone) and Collagen	ZnO nanoparticles and *Aloe vera* extracts	[86]
	Gelatin and collagen electrospinned onto a chitosan scaffold	*Lithosperm radix* (LR) extract	[87]
	Sodium alginate and collagen type-I hydrogel	Human umbilical cord mesenchymal stem cells (hUC-MSCs)	[88]
Stimulating ECM regeneration	Collagen/chitosan scaffold	Doxycycline	[89]
	Collagen scaffold	Silver nanoparticles conjugated with juglone	[90]
	Collagen type-I and tropoelastin scaffold	Not functionalized	[91]
	Bilayered collagen/chitosan—collagen–glycosaminoglycan scaffold	Not functionalized	[92]
	Collagen type-I and type-III crossed-fiber scaffold	Not functionalized	[93]
Particular case	Collagen/PLGA (poly-lactic-co-glycolic acid) scaffold	Glucophage (metformin)	[94]

## Data Availability

Not applicable.
